# Supplementary material for: A genome resource for Acacia, Australia’s largest plant genus
Source: PLoS One. 2022 Oct 14;17(10):e0274267. doi: 10.1371/journal.pone.0274267 (PMC9565413; doi:10.1371/journal.pone.0274267)
Supplement: S1 File — S1 A. NECAT assembly configuration details; file <acacia_config.txt>; S1 B. Merqury results and spectra plot; S1 C. Fixed topology trees used for OrthoFinder runs; S1 D. Visualisation of PhyloBayes chain_1.trace file in Tracer, showing log likelihood; S1 E. CAFÉ methods; S1 F. Whole Genome Duplication KDE plots; S1 G. Gene tree concordance factors of Leguminosae. (DOCX) [file pone.0274267.s001.docx]

**Supporting Information File S1**

**Contents:**

**S1 A NECAT assembly configuration details.**

**S1 B Merqury results and spectra plot.**

**S1 C Fixed topology trees used for OrthoFinder runs.**i) Fig S1: Fixed tree topology used as input for the Angiosperms OrthoFinder run.
ii) Fig S2: Fixed tree topology used as input for the Fabales OrthoFinder run.

**S1 D Visualisation of PhyloBayes chain_1.trace file in Tracer, showing log likelihood.**i) Fig S3: Visualisation of PhyloBayes chain_1.trace file in Tracer, showing log likelihood.

**S1 E CAFÉ methods.**

**S1 F Whole Genome Duplication KDE plots of K_s_ values.**

i) Fig S5. Kernel Density Estimate (KDE) plot of Ks distributions from one-to-one orthologs for Acacia pycnantha vs other Leguminosae taxa. The position of each peak on the x-axis (Ks) provides a proxy for the relative speciation time for each taxon pair.

ii) Fig S6. Kernel Density Estimate (KDE) plot of Ks distributions from anchor-pair paralogs for Acacia pycnantha and other Leguminosae taxa. The position of each peak on the x-axis (Ks) can provide a proxy for the relative time of gene or putative whole genome duplication for each taxon.

iii) Fig S7. Kernel Density Estimate (KDE) plot of Ks distributions from whole paranome for Acacia pycnantha and other Leguminosae taxa. The position of each peak on the x-axis (Ks) can provide a proxy for the relative time of gene or putative whole genome duplication for each taxon.

**S1 G Gene tree concordance factors of Leguminosae**i) Fig S8: Maximum likelihood phylogeny of 85 concatenated SCO genes using IQTree.

**S1 A. NECAT assembly configuration details; file <acacia_config.txt>**

PROJECT=acacia_necat

ONT_READ_LIST=read_list.txt

GENOME_SIZE=1000000000

THREADS=10

MIN_READ_LENGTH=3000

PREP_OUTPUT_COVERAGE=40

OVLP_FAST_OPTIONS=-n 500 -z 20 -b 2000 -e 0.5 -j 0 -u 1 -a 1000

OVLP_SENSITIVE_OPTIONS=-n 500 -z 10 -e 0.5 -j 0 -u 1 -a 1000

CNS_FAST_OPTIONS=-a 2000 -x 4 -y 12 -l 1000 -e 0.5 -p 0.8 -u 0

CNS_SENSITIVE_OPTIONS=-a 2000 -x 4 -y 12 -l 1000 -e 0.5 -p 0.8 -u 0

TRIM_OVLP_OPTIONS=-n 100 -z 10 -b 2000 -e 0.5 -j 1 -u 1 -a 400

ASM_OVLP_OPTIONS=-n 100 -z 10 -b 2000 -e 0.5 -j 1 -u 0 -a 400

NUM_ITER=2

CNS_OUTPUT_COVERAGE=30

CLEANUP=1

USE_GRID=false

GRID_NODE=0

GRID_OPTIONS=

SMALL_MEMORY=0

FSA_OL_FILTER_OPTIONS=

FSA_ASSEMBLE_OPTIONS=

FSA_CTG_BRIDGE_OPTIONS=

POLISH_CONTIGS=true

**S1 B. Merqury results and spectra plot**

| **Assembler** | **Completeness %** | **QV** | **Error rate** |
| --- | --- | --- | --- |
| **NECAT** | 88.4946 | 25.5 | 0.00280332 |

**i)** Completeness percentage refers to the ‘k-mer completeness’ of the assembly and is calculated via the number of non-error k-mers in the assembly divided by the number of non-error k-mers in the Illumina read set; the latter is assumed to accurately represent the true genome size. *ii)* QV, the ﻿assembly consensus quality value, is interpreted as a Phred quality score. *iii)* Error rate is calculated from k-mers found only in the assembly, ﻿which likely reflect base errors in the assembly consensus.


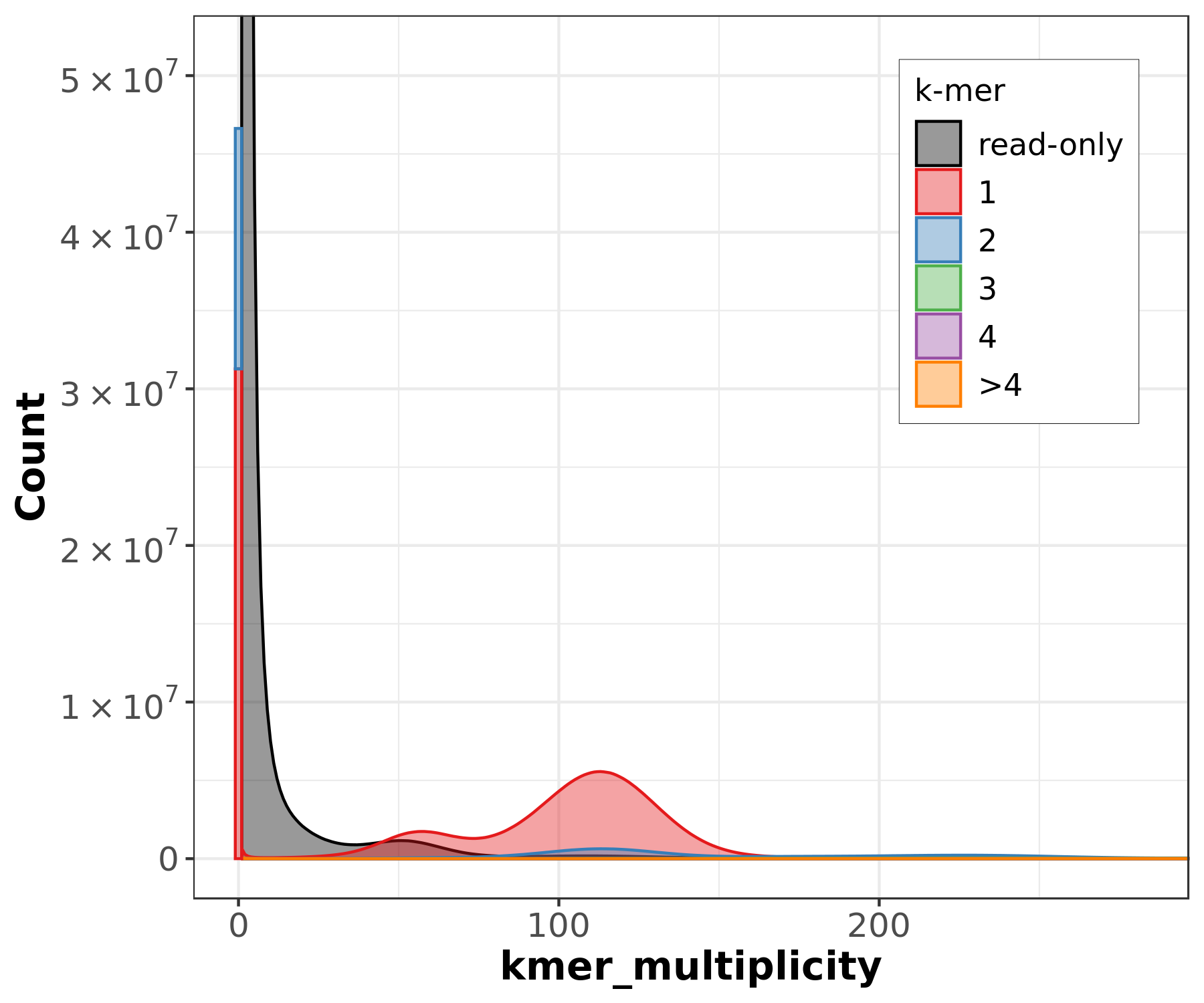


**ii)** A copy-number spectrum plot of k-mers found in the Illumina read set and the assembly, with the latter coloured based on their count in the assembly. The bar at the origin of the plot (zero multiplicity) shows k-mers absent from the read set (i.e., found only in the assembly); these likely represent base errors in the assembly consensus. k-mers found only in the read set are shown in black; those at low frequency (i.e., multiplicity) likely represent errors in the read set, whereas those at higher frequencies (e.g. black k-mers within the one or two-copy peaks at ~54x and ~109x multiplicity, respectively) indicate sequences missing from the assembly. The red peak at the 2-copy region (~ 109x multiplicity) represents homozygous two-copy k-mers that occur only once in the assembly, suggesting that purge-dups has largely succeeded in producing a pseudo-haploid assembly. The blue peak at the same region represents homozygous two-copy k-mers that still occur twice in the assembly, indicating that a small fraction of the assembly is still in diploid form.

**S1 C. Fixed topology trees used for OrthoFinder runs.**

1. **Fig S1.** Fixed tree topology used as input for the Angiosperms OrthoFinder run.

1. **Fig S2.** Fixed tree topology used as input for the Fabales OrthoFinder run.

**S1 D. Visualisation of PhyloBayes chain_1.trace file in Tracer, showing log likelihood.**


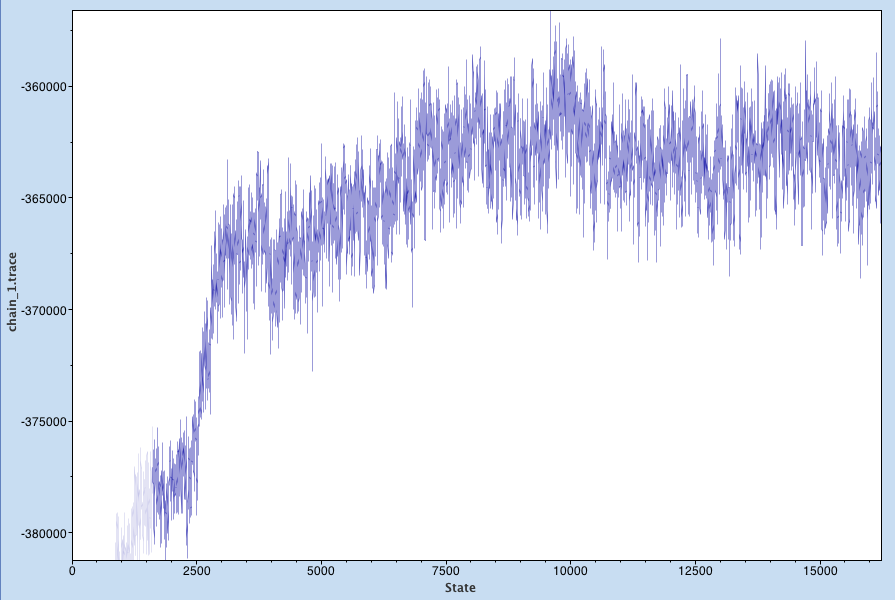


1. **Fig S3.** Visualisation of PhyloBayes chain_1.trace file in Tracer, showing log likelihood.

**S1 E. CAFÉ methods.**

Following recommendations from the CAFÉ authors (see CAFÉ tutorial webpage), the gene-count table (Supplementary File S2, Table S10) was filtered to produce two tables using the provided ‘clade_and_size_filter.py’ script; table T1, comprising gene families in which one or more species have ≥ 100 gene copies (59 families), and table T2, with the remaining genes families (19,856 families). The script also removes species-specific orthogroups (7,313 groups). Subsequently, T2 was analysed using the Gamma model implemented in CAFÉ, which allows a different evolutionary rate category for each gene family. A number of gamma rate categories (parameter -k) were tested (1 - i.e. a single rate for all families, 2, 3, and 4), and five CAFÉ runs were performed in each case to determine the k value with both the best fit to the data (highest log likelihood) and with convergence between runs. A k value of 2 was selected and a final CAFÉ run was performed using table T2 (estimated lambda value 0.0065630518393034, alpha value 1.9254615596352); after filtering for families with a gene at the tree root, 15,798 of 19,856 families were retained. Then, a CAFÉ run using Table T1 was performed using the Base model (i.e., a single gamma category for all families) and the lambda parameter previously estimated for T1 via the setting [-l 0.0065630518393034]; after filtering, 35 of 59 families were retained for analysis.

**S1 F. Whole Genome Duplication KDE plots**

**
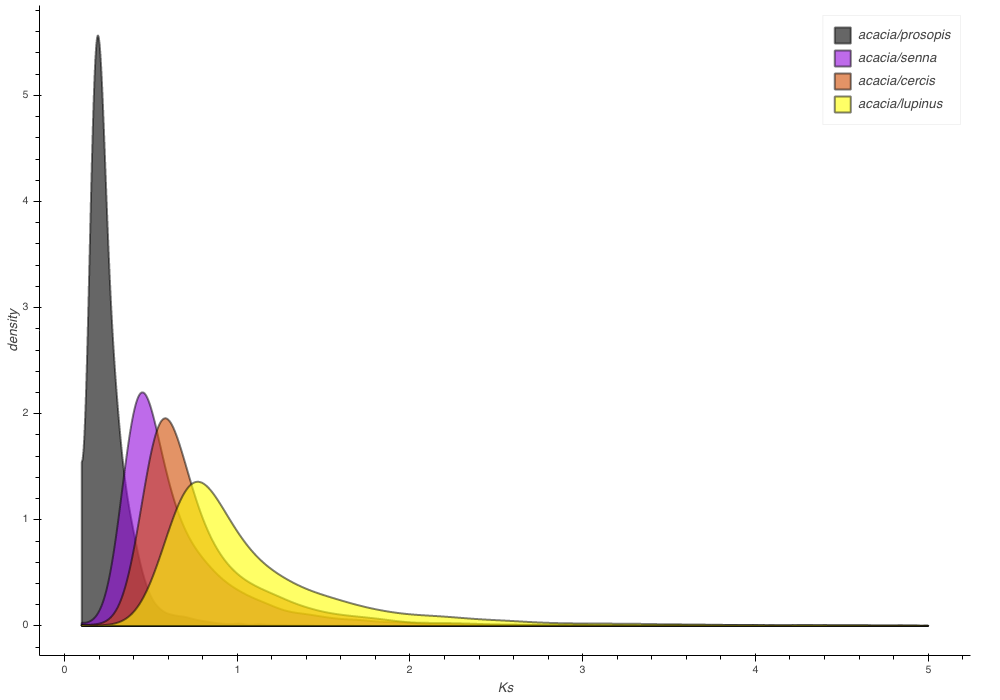
**

1. **Fig S4.** Kernel Density Estimate (KDE) plot of K_s_ distributions from one-to-one orthologs for *Acacia pycnantha* vs other Leguminosae taxa. The position of each peak on the x-axis (K_s_) provides a proxy for the relative speciation time for each taxon pair.


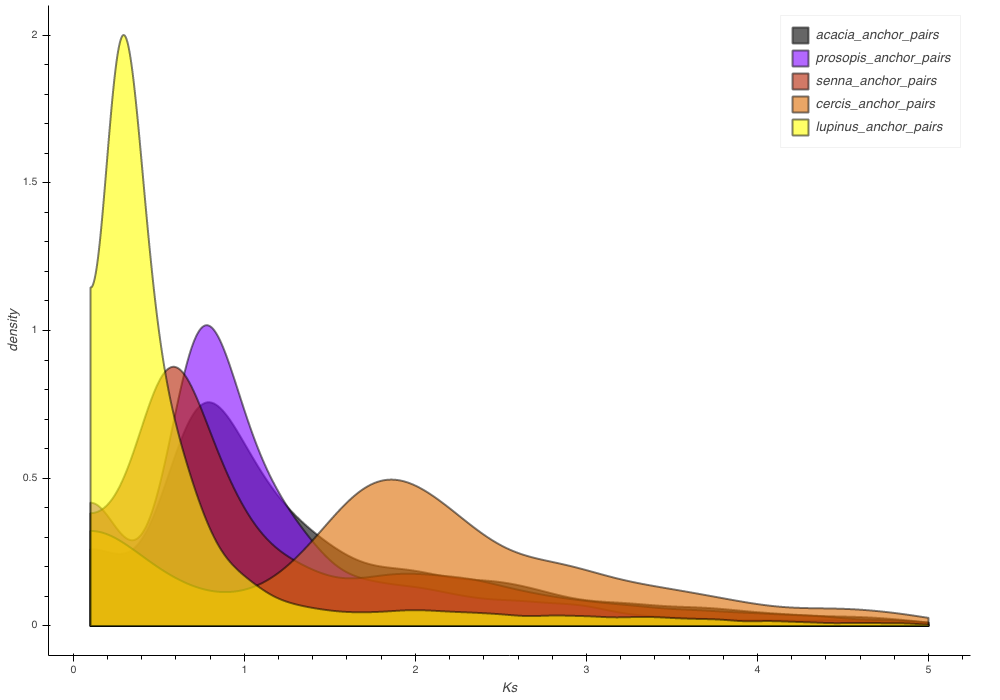


1. **Fig S5.** Kernel Density Estimate (KDE) plot of K_s_ distributions from anchor-pair paralogs for *Acacia pycnantha* and other Leguminosae taxa. The position of each peak on the x-axis (K_s_) can provide a proxy for the relative time of gene or putative whole genome duplication for each taxon.


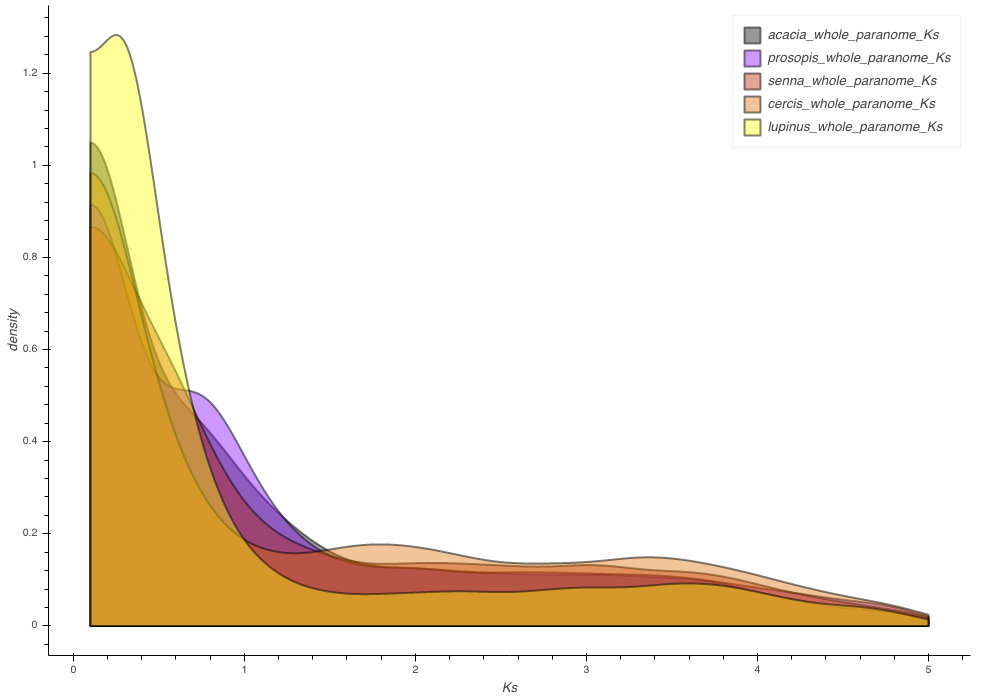


1. **Fig S6.** Kernel Density Estimate (KDE) plot of K_s_ distributions from whole paranome for *Acacia pycnantha* and other Leguminosae taxa. The position of each peak on the x-axis (K_s_) can provide a proxy for the relative time of gene or putative whole genome duplication for each taxon.

**S1 G. Gene tree concordance factors of Leguminosae**


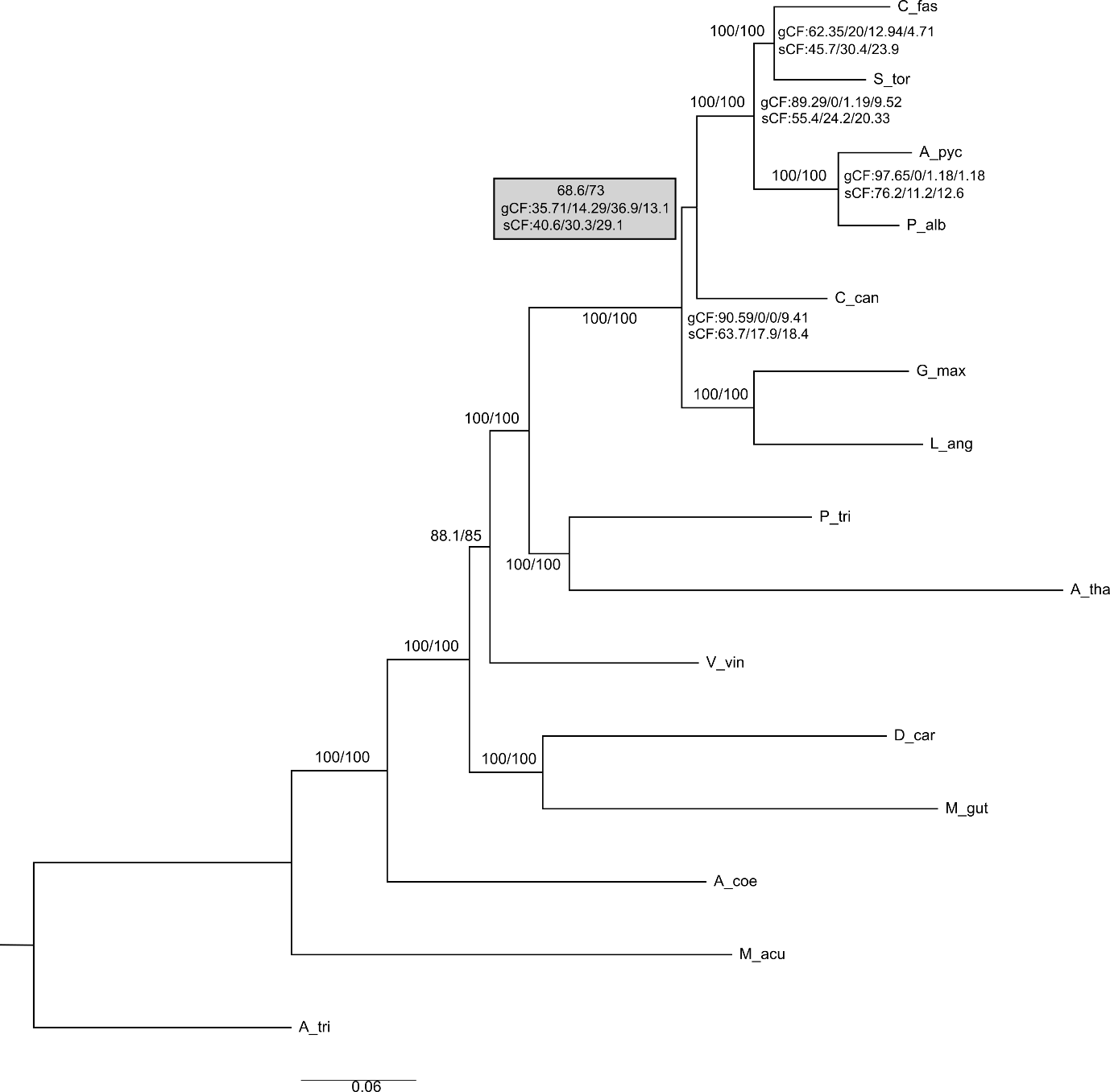


1. **Fig S7:** Maximum likelihood phylogeny of 85 concatenated SCO genes using IQTree. Numbers at nodes reflect sh-alrt/UFboot support values. Additionally, gene concordance factors (gCF:% genes concordant with present topology/% genes with topology NNI-1/%genes with topology NNI-2/% of genes with polytomy) and site concordance factors (sCF: site concordance factor%/site concordance factor for NNI-1%/site concordance factor for NNI-2%) are provided for Leguminosae.
