# Supplementary material for: A genome resource for Acacia, Australia’s largest plant genus
Source: PLoS One. 2022 Oct 14;17(10):e0274267. doi: 10.1371/journal.pone.0274267 (PMC9565413; doi:10.1371/journal.pone.0274267)
Supplement: Attachment — Submitted filename: response to reviews McLay Acacia.docx [file pone.0274267.s007.docx]

Dear Dr. Aceto,

Please find below a statement of the revisions performed on the manuscript, based on the comments of the two reviewers and yourself. Thank you for your efforts helping our manuscript get published.

Regards,

Todd McLay

1. Please ensure that your manuscript meets PLOS ONE's style requirements, including those for file naming.

*Manuscript checked for style and formatting.*

2. We note that the grant information you provided in the ‘Funding Information’ and ‘Financial Disclosure’ sections do not match.

When you resubmit, please ensure that you provide the correct grant numbers for the awards you received for your study in the ‘Funding Information’ section.

*Funding from the BioPlatforms – Genomics for Australian Plants is not a grant but a cofounding opportunity, and as such there is no grant number. This is stated in the in-text Funding Information and has been changed in the Financial Disclosure.*

3. We note that you have stated that you will provide repository information for your data at acceptance. Should your manuscript be accepted for publication, we will hold it until you provide the relevant accession numbers or DOIs necessary to access your data. If you wish to make changes to your Data Availability statement, please describe these changes in your cover letter and we will update your Data Availability statement to reflect the information you provide.

*The genome and associated data is under the existing BioProject accession PRJNA752212.* 4. Please include captions for your Supporting Information files at the end of your manuscript, and update any in-text citations to match accordingly. Please see our Supporting Information guidelines for more information: <http://journals.plos.org/plosone/s/supporting-information>.

*This is now in the correct place.*

5. Please review your reference list to ensure that it is complete and correct. If you have cited papers that have been retracted, please include the rationale for doing so in the manuscript text, or remove these references and replace them with relevant current references. Any changes to the reference list should be mentioned in the rebuttal letter that accompanies your revised manuscript. If you need to cite a retracted article, indicate the article’s retracted status in the References list and also include a citation and full reference for the retraction notice.

5. Review Comments to the Author

Please use the space provided to explain your answers to the questions above. You may also include additional comments for the author, including concerns about dual publication, research ethics, or publication ethics. (Please upload your review as an attachment if it exceeds 20,000 characters)

**Reviewer #1: Manuscript PONE-D-22-13018 presents a reference genome for the plant genus Acacia. The article is well-motivated, technically sound, and is presented in an interesting evolutionary context. I think it is suitable for publication in PLoS One in essentially its current form, and beyond that, will become a highly useful molecular resource.

I have a small number of very minor comments for the authors**:

Were any long-read assemblers besides NECAT tested with these data? If such tests were undertaken, and NECAT seemed like the best assembly, I would encourage the authors to share relevant observations in a supplement, since they might help guide the choices of researchers with similar long read datasets. Note, however, I will understand if the authors decline to do this. One sometimes tests assemblers only enough to determine that they are unlikely to produce the best assembly, and without optimizing enough for the purposes of a fair comparison… If so, understandable if they authors don’t want to share outcomes of relatively superficial tests of a package. However, if the authors did generate assemblies with other software somewhat carefully, the results are potentially interesting to many readers.

*We have added in information about the other assemblers we tested in the main text, and the results of those tests as Supp. Information File S2:Table S4.*

Figure captions do not seem to be visible in the version of the manuscript I downloaded, so I have not had an opportunity to review these. In particular, I think this led to some difficult interpreting Figure 3, especially the bottom two panels. For example, in the figure on the left, I wondered what the on-diagonal values meant – the number of orthogroups that an assembly shares with itself (is this the total number of genes, or some subset used in orthogroup analysis)? I also wondered if some normalization of these values could offer an interesting perspective? For example, rather than the numbers of genes, the proportion of genes in one species with orthologues in another…?

*Figure captions were in body of manuscript as per PLoS submission guidelines. These have been slightly modified in some cases for accuracy. For figure 3C (heatmap visualisations of orthogroup and ortholog species comparisons)*, *we would prefer to keep the current visualisation type (i.e. raw numbers), rather than normalising, as we believe these numbers provide useful insights into the genomes of each taxon. However, to better visualise these values in the left heatmap (orthogroups), we have rescaled the colour values so that the smallest value in our analysis corresponds to the darkest colour – this provides a greater range of values that can be visualised, and we hope makes the patterns much clearer. The corresponding code has been updated in the manuscript git repository. We also agree that the meaning of the on-diagonal values in both heatmaps were a bit obtuse, and we have added an explanation to the figure legend.*

Line 544 "a broad"?

*Added space as per reviewer.*

**Reviewer #2: McLay et al. reported their study on genome assembly and characterization of a Acacia species. Acacia genus and the studied species has significant values in many aspects. High quality genome assembly, annotation and characterization were generated and presented. Analytical methods, interpretation and discussion are all well.

Accept with a minor revision.**

minor comments:
1. line 287-28, in Abstract, please improve the confusing the English expression. You know, we can not generate long-read sequences using Illumina squences.
"We generated long-read sequences for A. pycnantha using Oxford Nanopore, 28 10x Genomics Chromium linked reads, and short-read Illumina sequences,"

*Changed sentence structure to improve clarity.*

2. line 68, "(e.g. [10]", sth lost here, at least one ")".

*Removed the ‘e.g.’, so now the sentence ends with the reference.*

3. line 346, I saw "Gbp", in line 347, I saw "Gb". Please follow a constant. Also, I read both "Kbp" and "Kb". Take care of them.

*Changed all occurrences to Gb and kb.*

4. line 356, "an N50 of 0.963 Gb"? please make it sure.

*Changed Gb to kb.*

5. Table 1. Do not use Captial letters for all words. And please provide units for some items such as "Contig N50".

*Changed the font and added units for terms.*

6. line 389-392, confusing sentence. "BLAST % identity" is read not a formal English expression.

*Changed to BLAST percent identity.*

**Additional changes**

1. Figure 3 and Figure 6 have had panel labels added/changed. Figure 3 was changed so the scale on the heatmap is more easily interpreted (no changes to data inputs).
   a. Fig3-orthofinder_figure-revisedJuly2022.eps
   b. Fig6-SRR1_phylogeny_PFAMdomain-revisedJuly2022.eps
2. A new supporting information file has been uploaded including a table addressing the comments of R1 regarding other genome assembly softwares used
   a. Fig6-SRR1_phylogeny_PFAMdomain-revisedJuly2022.xlsx
3. Reference list has been checked more closely for accuracy of references, no references were removed, references for assemblers have been added.
4. Position of supporting information details moved to end of manuscript, after reference list.
